# Identification of novel X-linked gain-of-function RPGR-ORF15 mutation in Italian family with retinitis pigmentosa and pathologic myopia

**DOI:** 10.1038/srep39179

**Published:** 2016-12-20

**Authors:** Francesco Parmeggiani, Vanessa Barbaro, Katia De Nadai, Enrico Lavezzo, Stefano Toppo, Marzio Chizzolini, Giorgio Palù, Cristina Parolin, Enzo Di Iorio

**Affiliations:** 1 Department of Biomedical and Specialty Surgical Sciences, University of Ferrara, Ferrara, 44121, Italy; 2 Fondazione Banca degli Occhi del Veneto, Venice, 30174, Italy; 3 Center for Retinitis Pigmentosa of Veneto Region, ULSS 15 Alta Padovana, Camposampiero, 35012, Italy; 4 Department of Molecular Medicine, University of Padova, Padova, 35121, Italy

## Abstract

The aim of this study was to describe a new pathogenic variant in the mutational hot spot exon ORF15 of retinitis pigmentosa GTPase regulator (RPGR) gene within an Italian family with X-linked retinitis pigmentosa (RP), detailing its distinctive genotype-phenotype correlation with pathologic myopia (PM). All members of this RP-PM family underwent a complete ophthalmic examination. The entire open reading frames of RPGR and retinitis pigmentosa 2 genes were analyzed by Sanger sequencing. A novel frame-shift mutation in exon ORF15 of RPGR gene (c.2091_2092insA; p.A697fs) was identified as hemizygous variant in the male proband with RP, and as heterozygous variant in the females of this pedigree who invariably exhibited symmetrical PM in both eyes. The c.2091_2092insA mutation coherently co-segregated with the observed phenotypes. These findings expand the spectrum of X-linked RP variants. Interestingly, focusing on Caucasian ethnicity, just three RPGR mutations are hitherto reported in RP-PM families: one of these is located in exon ORF15, but none appears to be characterized by a high penetrance of PM trait as observed in the present, relatively small, pedigree. The geno-phenotypic attributes of this heterozygosity suggest that gain-of-function mechanism could give rise to PM via a degenerative cell-cell remodeling of the retinal structures.

Retinitis pigmentosa (RP; OMIM 268000) represents the most frequent hereditary disease of the retina, affecting approximately one in 3000–4000 individuals. RP is commonly inherited as monogenic autosomal-dominant (about 30–40% of cases), autosomal-recessive (50–60%) or X-linked (5–15%) trait, even if digenic and mitochondrial patterns are also described. This retinopathy can be a part of complex phenotypic disorder (syndromic RP), but in the majority of the patients it is an isolated disease (simplex RP)^1^,^2^. RP causes an irreversible photoreceptor degeneration, which results in highly variable clinical consequences dependent on the prevailing damages of rods or cones, age of onset, and rate of progression. Although the typical form of RP is invariably related to the primary rods deterioration in both eyes and its early symptoms include night blindness and/or visual field loss, the genetic etiology of RP is exceptionally heterogeneous. In fact, this monogenic disease can be cause by a lot of known and unknown mutations, whose unpredictable levels of penetrance and expressivity lead to a very high complexity in the definition of genotype-phenotype correlation in several RP cases^3^,^4^,^5^,^6^. In particular, X-linked RP is thought to become evident only in the mutated male members of the affected families, but variable disease-related phenotypes can be also observed in some carrier females (from asymptomatic to severe retinal degeneration in one or both eyes), mimicking a Mendelian dominant transmission^7^,^8^,^9^. To date, six genetic loci have been mapped on human X-chromosome (see the Retinal Information Network [RetNet] at https://sph.uth.edu/retnet/ and the Human Gene Mutation Database [HGMD] at http://www.hgmd.cf.ac.uk) but, according to the linkage analyses, RP-associated mutations are recognized in two main genes: retinitis pigmentosa GTPase regulator (RPGR or RP3; OMIM 312610) and retinitis pigmentosa 2 (RP2; OMIM 312600). In fact, the majority of genetically solved X-linked RP can be explained by causative mutations in RPGR and RP2 genes accounting, respectively, for 75–80% and 10–15% of the genetically-solved cases of the disease^1^,^5^,^6^,^7^,^8^,^9^,^10^,^11^. In this study, we report a Caucasian family with a novel pathogenic sequence alteration in the mutational hot spot exon ORF15 of RPGR gene underlying X-linked RP and pathologic myopia (PM). Because of the distinctive genotype-phenotype correlation, characterized by a symmetrical presence of PM in both the retinas of all heterozygous female carriers, a speculative model of X-linked gain-of-function mutation with a high penetrance of the myopic trait is proposed.

## Results

**Figure 1:**
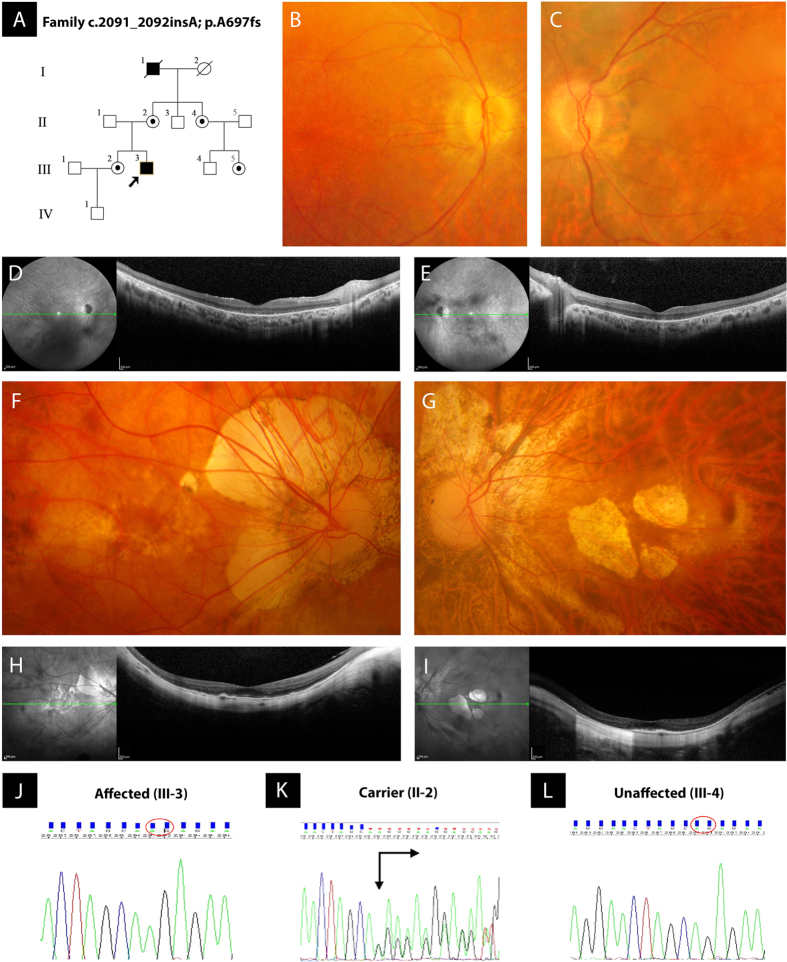
Pedigree of a non-consanguineous family with X-linked retinitis pigmentosa (RP) and pathologic myopia (PM), retinal imaging, and novel frame-shift mutation identified in exon ORF15 of RPGR gene. (**A**) Pedigree of the RP-PM family. Black squares (males) represent individuals affected by RP. Dotted circles (females) represent individuals affected by PM. Unaffected individuals are not shaded. Black lines indicate deceased individuals. Each generation is identified by a Roman numeral on the left (from I to IV), and each individual within the generation is identified by Arabic numerals next to the symbols. The arrow marks the proband. (**B**,**C**) Color fundus photographs of the posterior pole of the proband III:3 bilaterally show the typical aspect of RP, characterized by optic disc pallor, attenuated retinal vessels, macular dystrophy, and degenerative changes of the retinal pigment epithelium with an irregular visualization of the peri-papillary choroidal vasculature. (**D**,**E**) Spectral-domain optical coherence tomographies of the macula of the proband III:3 document the degenerative changes of retinal layers in both eyes, revealing the structural damages of both inner segment ellipsoid band and photoreceptor outer segment. (**F**,**G**) Color fundus photographs of the posterior pole of the obligate carrier II:2 bilaterally exhibit the features of severe PM, characterized by titling of the optic disc with an extensive peri-papillary atrophic crescent, straightened and stretched retinal vessels, patchy chorioretinal areas of macular atrophy and dystrophy, and diffuse degenerative changes of the retinal pigment epithelium with a marked visualization of the choroidal vasculature. (**H**,**I**) Spectral-domain optical coherence tomographies of the obligate carrier II:2 confirm the phenotypic signs of PM-related macular degeneration in both eyes, typically evidencing a myopic staphylomatous configuration of the posterior pole. (**J**–**L**) Representative sequence chromatograms of the hemizygous male proband III-3, heterozygous obligate female carrier II-2, and unaffected proband’s maternal male cousin III-4 are illustrated.

**Figure 2:**
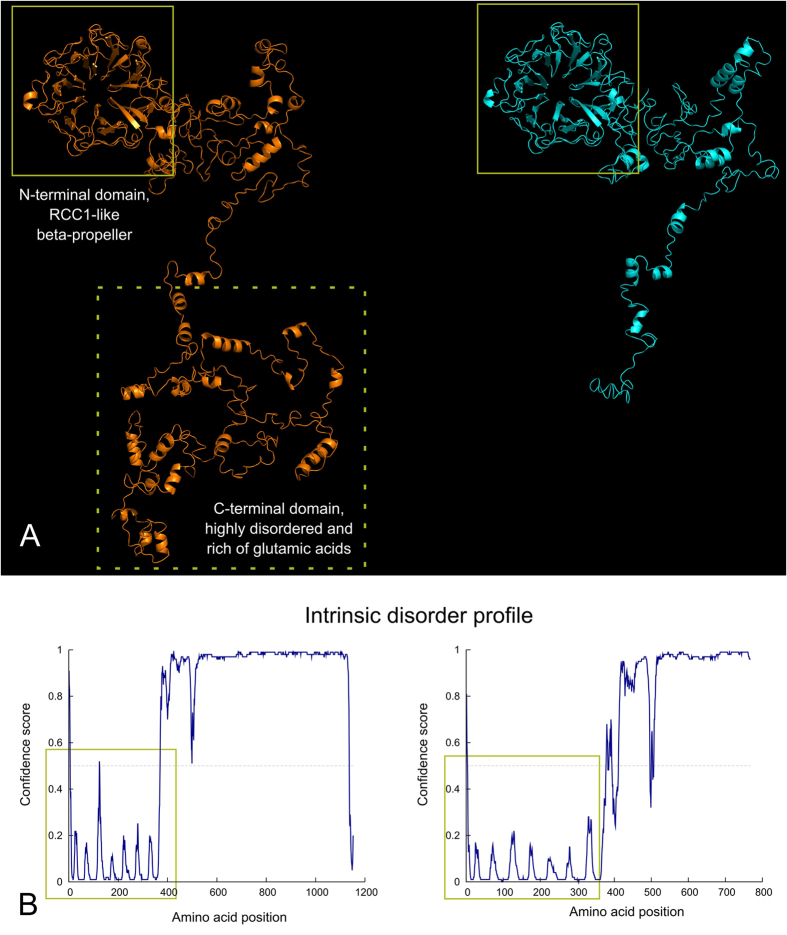
Structural comparison between wild type and mutant RPGR protein and intrinsic protein disorder prediction. (**A**) Wild type (left) and mutant (right) RPGR protein models are shown: while the N-terminal RCC1-like domain is conserved (highlighted by yellow boxes), the frame-shift mutation induces the loss of a long C-terminal domain highly rich of glutamic acid residues (dotted box), resulting in a much shorter protein. (**B**) The intrinsic disorder profile shows how the conserved N-terminal domain is extremely ordered (yellow boxes) in contrast with the remaining portion that does not possess an intrinsic structure. Seven spikes corresponding to connecting loops of the seven blade-shaped beta sheets of the beta-propeller structure are visible in the profile. Please note that the X-axis scale is different because of the length of the proteins.

The non-consanguineous RP-PM family of this study included 13 individuals in a four-generation pedigree (Fig. 1A) originating from the North-East of Italy. Eleven family members were clinically and genetically investigated: one proband showing typical RP (III:3), four carriers of severe PM (II:2, II:4, III:2 and III:5), and six unaffected individuals including three male spouses (II:1, II:3, II:5, III:1, III:4 and IV:1). The 34-year-old male proband (III:3) experienced increasing night blindness and photophobia since childhood and, during adolescence, a progressive loss of his peripheral vision. Starting from 13 years of age, he reported the bilateral onset of mild-to-moderate myopic refractive error and, during his second decade of life, central visual deficiency with significant reduction of best-corrected visual acuity (BCVA). In both eyes, this young adult patient had mild polar posterior subcapsular cataract. His bilateral fundus appearance included an extensive vitreous degeneration, moderate optic disc pallor, attenuated retinal vessels, macular dystrophy, degenerative changes of the retinal pigment epithelium (RPE) with an irregular visualization of the peri-papillary choroidal vasculature, and mid-peripheral bone spicule-shaped pigment deposits (Fig. 1B,C). In the macula of both eyes, moderate retinal thinning along with structural changes of both inner segment ellipsoid band and photoreceptor outer segment were observed by means of spectral-domain optical coherence tomography (SD-OCT) (Fig. 1D,E). Computerized visual field examination bilaterally documented the presence of a severe concentric narrowing of the vision area, and the tracings of full-field electroretinography (ff-ERG) were markedly reduced. He had no audiometric abnormalities that could be related to Usher’s syndrome, as well as specific systemic disorders suggestive of other syndromic RPs. The familial history revealed that his deceased maternal grandfather (I:1) had suffered from typical RP too, as documented by former ophthalmologic reports. Symmetrical PM, without any pathognomonic sign of RP at the ophthalmoscopic examination, was diagnosed in both eyes of all female relatives of the proband, i.e. mother (II:2), sister (III:2), maternal aunt (II:4), and maternal cousin (III:5). These patients were reported to bilaterally have high myopic refractive error since the age of 10 years, with variable reduction of BCVA during their second decade of life. There were also slow progressions of both nyctalopia and peripheral vision deficiency with rather different levels of symptoms severity. With no exception, all these four women had high myopia in each of their eyes, and the axial length was always greater than 29.5 millimeters. Slight nuclear cataract, with small polar posterior subcapsular opacity, was bilaterally present in the two II-generation females affected by PM (II:2 and II:4). In both eyes of the proband’s mother (II:2), funduscopy revealed the typical structural and degenerative changes characterizing the severe PM: tilted optic disc with a wide peri-papillary atrophy, macular chorioretinal damages, extensive RPE-changes with a marked visualization of the choroidal vasculature, straightened and stretched retinal vessels (Fig. 1F,G). In both eyes, SD-OCT examination confirmed the above-described signs of myopic maculopathy, pointing out the presence of moderate retinal thinning associated with macular posterior staphyloma (Fig. 1H,I). Computerized visual field exam bilaterally documented blind spot enlargement, irregular central reduction of retinal sensitivity, and several myopic arcuate-like defects in the mid-peripheral sectors. In both eyes, ff-ERG amplitude was significantly decreased. In the other three female members of this RP-PM family, ophthalmoscopic, tomographic, perimetric and electroretinographic findings invariably resembled those detected in the proband’s mother, leading to the same diagnosis of symmetrical PM with remarkable visual field defects and ff-ERG decays in both eyes of patients II:4, III:2 and III:5. These diagnostic examinations were also conducted on the six asymptomatic males of this pedigree (II:1, II:3, II:5, III:1, III:4 and IV:1), without detecting any specific and/or significant ocular disorder. Spanning all exons of RPGR and RP2 genes, the direct sequencing of PCR products of the proband with RP revealed the hemizygous status for a novel causative frame-shift mutation in exon ORF15 of RPGR gene (c.2091_2092insA [g.ORF15+338_339insA]; p.A697fs [p.ORF15+A112fs]) (Fig. 1J). Both the proband’s mother (obligate carrier; Fig. 1K) and the other three female carriers with degenerative PM were found to be heterozygous for this RP-related variant. This mutation was neither observed in the proband’s male cousin (Fig. 1L) nor in the other five unaffected members of this RP-PM family nor in one hundred ethnically matched control individuals. The disease-causing mutation c.2091_2092insA coherently co-segregated with the phenotypic conditions of all family members, confirming the presence of X-linked inheritance pattern. A summary of clinical and genotypic findings is provided in Table 1. Finally, the structural comparison between the models of wild type and mutant RPGR protein is shown in Fig. 2.

## Discussion

The genetic etiology of X-linked RP is continuously growing and widely changeable across the ethnic groups. It is characterized by high allelic heterogeneity and variable phenotypic expressivity among both affected males and carrier females who, in particular, may or may not have symptoms and clinical signs^9^. In the course of our routine geno-phenotyping practice conducted on RP probands with a provisional diagnosis of X-linked inheritance and his relatives, we have studied a non-consanguineous Italian family with one male affected by typical RP and four females suffering from bilateral high PM, identifying a novel frame-shift pathogenic variant in the mutational hot spot exon ORF15 of RPGR gene.

RPGR gene gives rise to at least 12 alternatively spliced isoforms. Some of its transcripts are ubiquitously expressed, while other ones are tissue-specific containing extra exons that elongate or truncate the protein^12^,^13^. RPGR gene product is necessary for the normal function of retinal photoreceptors. Mutations in RPGR are associated with X-linked RP, as well as with X-linked cone dystrophy, cone-rod dystrophy, and an atrophic form of macular degeneration^14^. RPGR is a cilia-centrosomal protein, involved in both regulation of cilia function and facilitation of proteins trafficking along the photoreceptor cilium (intraflagellar transport), whose genotypic defects result in RP phenotype^15^,^16^,^17^. Although the number of RPGR protein isoforms expressed in the human retina is still unclear, two isoforms are the most important for RP genetics: RPGR^1–19^ (amino acids 815; exons 1–19) and RPGR^ORF15^ (amino acids 1152; exons 1–15 and part of intron 15). Both isoforms share exons 1 through 15 and the N-terminus part containing a domain homologous to the RanGEF regulator of chromosome condensation 1 (RCC1), whereas only RPGR^ORF15^ has a glu-gly-rich C-terminal domain^7^,^12^,^13^,^17^. The N-terminal domain of RPGR is a beta-propeller structure made of seven blade-shaped beta sheets conserved in both wild type and mutant proteins. RCC1 is a nuclear protein that binds to both nucleosomes and double-strand DNA and promotes the exchange between GDP and GTP on Ran (a Ras-like protein), thus regenerating its activity. For this reason, RPGR is predicted to have a GEF activity (guanine nucleotide exchange factor), but this function should be exerted in the connecting cilium of rod and cones, where the protein localizes. As shown in the protein structure model predictions, the herein described c.2091_2092insA frame-shift mutation in exon ORF15 of RPGR gene causes the loss of a 455 amino acids long C-terminal domain, which is replaced by 71 new residues resulting in a truncated protein (p.A697fs). The lost domain is intrinsically disordered and flexible, suggesting a possible role in protein-protein interactions, and contains numerous glutamic acid residues which confer a pronounced negative charge to the domain. While its function is currently unknown and difficult to predict, its structural and negatively charged features could suggest a possible implication in binding positively charged substrates, for example acting as a scaffold for membrane-bound protein complexes (often positively charged in order to interact with membrane phospholipids) or providing a reservoir of cytoplasmic calcium ions.

Considering that p.A697fs originates from a codon located in ORF15+338_339 position, the proband’s phenotype agrees with previously reported data. In fact, this novel frame-shift mutation causes a typical rod-cone dystrophy as characteristically occurred in patients with RPGR-related X-linked RP who harbor ORF15 variants upstream of codon 445^18^,^19^. Moreover, the co-segregation analysis of this Caucasian family suggests the occurrence of a distinctive X-linked genotype-phenotype correlation between RP and PM, in which a possible complete penetrance of PM trait cannot be ruled out even considering the numerical pedigree’s limitation. In fact, just four female carriers suffered from bilateral myopic chorioretinal degenerations but all of them were heterozygous for ORF15-c.2091_2092insA, indicating that PM could represent the phenotypic expression of RP-related mutant heterozygosities located in various exons of RPGR gene, as previously observed in several pedigrees of both Asian and Caucasian descents^20^,^21^,^22^,^23^,^24^,^25^,^26^,^27^,^28^. Focusing on the mutational hot spot exon ORF15 of RPGR gene^10^,^19^,^29^,^30^, RP-PM mutations have been hitherto reported exclusively in Asian pedigrees^21^,^23^,^26^, with the exception of the c.2543del variant that has been recently discovered in a family of Caucasian Czech origin^28^. In four of these seven RP-PM families with different frame-shift mutations in exon ORF15 of RPGR gene, the variable occurrence of PM among the heterozygous female carriers or between the eyes of the same patients indicates the presence of an incomplete penetrance of this degenerative trait^21^,^26^,^28^. In fact, an unequivocal PM phenotype, characterized by high myopic refractive error along with myopic fundus appearance, has been detected in both eyes of all RPGR-ORF15 mutant pedigree’s females exclusively in three Asian families^21^,^23^, and just one of these included more than two heterozygous carriers with PM^21^ resembling those genealogical features reported for the first time by ours in the present Caucasian family. Even if the categorization of mutant alleles into predicted-nulls versus predicted-translated proteins may not correspond to the presence or absence of protein bio-translation inside retina, each of these two heterozygosities consists of a single nucleotide insertion in exon ORF15 of RPGR gene, underlying bilateral and severe PM in all female carriers regardless of their ethnicities^21^. Interestingly, both the already known g.ORF15+753_754 insG^21^ and our novel g.ORF15+338_339insA result in a truncated protein without the typical configuration of the normal C-terminal domain rich in glutamic acid residues. Notionally, all the diverse retinal changes of the heterozygous females with an X-linked RP frame-shift mutation in RPGR gene should be suspected to be due to a peculiar truncation of RPGR protein^21^,^23^,^26^,^28^,^31^, and each of these carriers represents a mosaic of photoreceptors expressing either the wild type or the mutant allele. Although further studies on the mechanism underlying gain-of-function damages of RPGR mutants are necessary to elucidate molecular functions and interactions at the photoreceptor cilium, in cases of polypeptidic biopolymers, such as RPGR, the presence of cells containing truncated proteins can reliably result in more severe phenotypes than those observed in heterozygous carriers with loss-of-function mutants. This possibility may be also pondered reviewing the data recently reported by Sun *et al*., who have described a Chinese, 5-year-old, heterozygous female carrier of RPGR c.139_140insTCTGC mutation as a proband with early-onset PM^32^. In the vertebrates’ retinas, cell-cell interactions are able to transfer toxic compounds from one cell to an adjacent other via gap junction channels and/or extracellular routes, remodeling the retinal structures in variable patterns of photoreceptors degeneration^33^,^34^,^35^. In this pathogenic context, the bilateral PM occurrence in all our Caucasian heterozygous carriers of the RPGR-ORF15 c.2091_2092insA mutation indicates that this juvenile-onset chorioretinal damage may be reliably related to how the two photoreceptor clusters interact by themselves, similarly to what has been already described in several females belonging to Asian pedigrees^21^,^23^. Retinal changes resulting from the cohabitation of wild type and mutant photoreceptors will also depend on lyonization, the process by which happens the random inactivation of one of the X-chromosomes in the cells of females to compensate the presence of double X gene dose. Considering the particular mode of PM-trait expressivity of RPGR-ORF15 heterozygosities, a skewed X-chromosome inactivation should be not necessarily associated with the phenotypic severity of myopia in our female carriers. However, in these patients, the evident p.A697fs-related changes in the intrinsically disordered C-terminus of the protein indicate the possible detrimental role of RPGR for the development of myopic retinal degeneration. The above-described pathogenesis of PM appears to be also indirectly supported by different experimental findings in canine and murine RPGR exon ORF15 mutations. The data obtained from both these animal models have indicated a rational theory to explain the high phenotypic variability resulting from RPGR-ORF15 mutations, suggesting the possibility that a subset of human RPGR mutations can behave as dominant gain-of-function alleles^36^,^37^. In particular, Hong *et al*. have observed that the expression of a truncated RPGR mutant in the retina of transgenic mice causes rapid photoreceptor degeneration in both wild type and RPGR null background, highlighting that the mutant induces a deleterious effect beyond what could be accounted just by the loss of RPGR function and, finally, hypothesizing that an abnormal truncated RPGR protein could be able to interfere with intraflagellar transport and disc morphogenesis at the photoreceptor cilium^37^.

From time to time, semi-dominant forms of X-linked RP have been identified analyzing both RPGR and RP2 genes of different ethnic origins^7^,^9^,^11^,^24^,^28^,^31^,^38^,^39^,^40^,^41^. The majority of these disease-causing mutations are located in exon ORF15 of RPGR gene, and their propensity to mimic an autosomal dominant inheritance is often owing to the presence of female carriers with PM in the pedigree^9^. Starting from the first analytical report on the refractive errors of RP patients, mild-to-high myopia has been described as a very frequent feature in the majority of affected family members with X-linked disease^42^. Moreover, in families without male-to-male RP transmission, myopia, associated with highly variable degenerative chorioretinal changes, is present in many women^20^,^21^,^22^,^23^,^24^,^25^,^26^,^27^,^28^,^31^,^38^,^39^,^40^. Even if the skewed X-chromosome inactivation should be the key factor implicated in the wide inter-individual and inter-ocular diversity of the expression of these myopic traits^11^,^24^, other unknown genetic modifiers could contribute to the phenotypic heterogeneity of PM in heterozygous female carriers of X-linked RP mutations. Until the genotype-phenotype correlations between X-linked RP and PM will be unclear, RPGR and RP2 sequencing and genetic biobanking should be recommended in the pedigrees with two or more X-linked women affected by symmetric or asymmetric PM where potential male probands with RP is absent or not available. Comprehensive recognition of X-linked gain-of-function mutations in RP-PM families and systematic assessment of their genotype-phenotype correlations are necessary to facilitate appropriate genetic counseling and future gene-based treatments. The study of new speculative models of disease’s expressivity in mutant heterozygous female carriers should be considered to open a wide and strong opportunity in the gene replacement therapy for RPGR-ORF15-associated retinal degeneration, due to the fact that by silencing just the anomalous gene product one could re-establish a normally enough phenotype in the patients.

## Methods

### Clinical evaluation

All the participants to this study were clinically examined at the Center for Retinitis Pigmentosa of the Veneto Region of the ULSS 15 Regional Hospital of Camposampiero (Italy). The diagnosis of typical RP was based on characteristic disease’s symptoms (such as night blindness and/or progressive loss of peripheral vision), accompanied by pathognomonic bilateral alterations in visual field, ophthalmoscopy, and ff-ERG. Moreover, pure tone audiometry was evaluated to rule out Usher’s syndrome; other syndromic RPs, such as Bardet-Biedl syndrome, Laurence-Moon disease, Alstrom disease, Refsum disease and so on, were also excluded. The diagnosis of PM was defined as myopic refractive error (spherical equivalent) more than 6 negative diopters or axial eye length >26.5 millimeters. All individuals had a complete ophthalmic examination, including refraction, BCVA measured in decimal equivalents by standard logarithmic chart at a test distance of three meters, slit lamp biomicroscopy and lens assessment performed according to the Lens Opacities Classification System III (LOCS III)^43^, applanation tonometry, funduscopy after pupil dilatation, axial eye length measurements using the non-contact partial coherence laser interferometry of IOL Master Version 5.0 (Carl Zeiss Meditec, Jena, Germany), computerized static threshold visual field exam using the central 30-2 SITA standard strategy and III-white stimulus of Humphrey field analyzer (Carl Zeiss, Oberkochen, Germany), macular SD-OCT using Spectralis Plus system (Heidelberg Engineering, Inc, Heidelberg, Germany), and ff-ERG recorded by Retimax Plus system (Costruzioni Strumenti Oftalmici, Florence, Italy) according to the standards of the International Society for Clinical Electrophysiology of Vision (ISCEV)^44^. In addition, one hundred unrelated healthy Italian individuals were utilized as control group. Controls were randomly selected from blood donors who did not report any personal or familial history of hereditary retinopathies, and voluntarily participated in this study after undergoing complete ophthalmologic visit to confirm that they were not affected by RP and other major eye diseases. Informed consents were obtained from all patients and controls (or their legal guardians) before their participation in the present clinic-genetic investigation. Research protocols adhered to the tenets of the Declaration of Helsinki and were approved by the local institutional Ethics Committee of the University of Padova (DIIO_EPPR_P13_03).

### Molecular genetic evaluation

All the participants to this study were genetically screened at the Department of Molecular Medicine of the University of Padova, Italy. Genomic DNA was obtained from peripheral blood samples using an automated DNA extractor (Roche, Mannheim, Germany) following the manufacturer’s instructions. The amplification PCRs for RPGR and RP2 genes were performed in 33 or 35 μl reaction containing ReadyMix^TM^ REDTaq^R^ PCR (Sigma, Seelze, Germany), water and primers forward and reverse mix 10 μM; under conditions of initial denaturation for 5 min at 95 °C, 45 cycle of amplification with 94 °C for 30 s, 58 °C/50 °C for 30 s, 72 °C for 1 min and 72 °C final extension for 5 min. The entire open reading frame of RPGR and RP2 genes, including all exons and flanking intronic sequences, was analyzed. Nineteen primer sets for RPGR-promoter exons 1–19, four primer sets for RPGR ORF15 exon, and five primer sets for RP2 exons were synthesized by Invitrogen (Invitrogen, Grand Island, NY) or Sigma (Sigma, Seelze, Germany). The primer sets amplify each exon and at least 60 bp of intronic sequence on either end. All primer pairs for RPGR and RP2 genes were designed from the genomic sequences (Gen-Bank accession numbers NM_001034853 [RPGR] and NT_006915 [RP2]) using Primer3 software (available at http://bioinfo.ut.ee/primer3/; accessed 25 March 2016); primers sequences are available upon request. The mutation screening was performed by directly sequencing RPGR and RP2 genes using Sanger method. Products were sequenced by using reagents BigDye^R^ Terminator v3.1 and automated sequencers Genetic Analyzer 3130xl according to the manufacturer’s protocols (Applied Biosystems, Carlsbad, CA). Sequences were aligned using SeqScape^R^ v2.5 (Applied Biosystems, Carlsbad, CA). The pathogenicity of unreported variant was established by the following criteria: (1) co-segregation in the proband’s family, (2) absence in 100 healthy control individuals after screening by Sanger sequencing, (3) absence in the single nucleotide polymorphism database (information available at https://sph.uth.edu/retnet/ and http://www.hgmd.cf.ac.uk; accessed 29 July 2016), and (4) prediction to result in a truncated protein or in a peptide with a change in inter-species highly conservative amino acids. The amino acid sequences of both wild type (RefSeq NM_001034853.1) and mutant RPGR ORF15 have been processed with the I-TASSER web server^45^, which builds structural models starting from templates of similar proteins. The same sequences were analyzed with the DISOPRED module of the PSIPRED web server^46^. Models were visualized with PyMol (The PyMOL Molecular Graphics System, Version 1.8 Schrödinger, LLC).

## Additional Information

**How to cite this article**: Parmeggiani, F. *et al*. Identification of novel X-linked gain-of-function RPGR-ORF15 mutation in Italian family with retinitis pigmentosa and pathologic myopia. *Sci. Rep.* **6**, 39179; doi: 10.1038/srep39179 (2016).

**Publisher's note:** Springer Nature remains neutral with regard to jurisdictional claims in published maps and institutional affiliations.

## Figures and Tables

**Table 1:** Summary of clinical and genotypic findings (Family RPGR-ORF15 c.2091_2092insA; p.A697fs).

ID/Sex/Age (Y)	Refraction (ESD) OD/OS	BCVA (SE) OD/OS	LOCS III Grading (NO/NC/C/P) OU	IOP (mmHg) OD/OS	Axial Length (mm) OD/OS	Visual Field MD (decibel) OD/OS	ff-ERG Amplitude (μVolts) OD/OS	Genotype/Phenotype
II:1/M/58	−0.50/−0.25	20/20/20/20	0/0/0/0	16/16	24.12/24.26	−0.55/−0.39	242.3/244.8	normal/normal
II:2/F/54	−14.00/−16.75	20/32/20/40	1/1/0/1	15/16	31.28/32.79	−15.56/−19.04	121.4/124.3	HET-FSM/PM
II:3/M/52	+1.50/+1.75	20/20/20/20	0/0/0/0	18/17	23.84/23.88	−0.62/−0.71	249.9/246.8	normal/normal
II:4/F/50	−13.50/−15.00	20/32/20/32	1/1/0/1	17/17	30.23/31.51	−16.03/−18.86	135.7/137.9	HET-FSM/PM
II:5/M/55	−1.75/−1.50	20/20/20/20	0/0/0/0	16/16	24.94/24.89	−0.46/−0.37	239.6/238.1	normal/normal
III:1/M/35	+0.25/+0.25	20/20/20/20	0/0/0/0	18/18	24.03/23.92	−0.14/−0.09	251.7/247.3	normal/normal
III:2/F/34	−12.75/−14.25	20/25/20/32	0/0/0/0	16/15	30.04/31.12	−20.89/−21.71	129.9/128.2	HET-FSM/PM
III:3/M/31 (*)	−5.50/−5.25	20/63/20/63	0/0/0/1	14/14	25.78/25.75	−29.54/−31.00	19.2/21.3	HEM-FSM/RP
III:4/M/27	−2.50/−2.00	20/20/20/20	0/0/0/0	18/18	24.33/24.35	−0.39/−0.31	251.7/249.2	normal/normal
III:5/F/25	−13.25/−12.50	20/25/20/25	0/0/0/0	17/17	30.12/29.74	−15.09/−14.94	141.9/145.3	HET-FSM/PM
IV:1/M/8	+1.50/+1.75	20/20/20/20	0/0/0/0	18/18	23.17/23.12	−0.08/−0.15	255.3/258.2	normal/normal

ID, Identification code of patients; Y, Years; M, Male; F, Female; (*), proband; ESD, Equivalent Spherical Diopters; OD, Oculus Dexter; OS, Oculus Sinister; BCVA, Best Corrected Visual Acuity; SE, Snellen Equivalent; LOCS III, Lens Opacities Classification System III; NO, Nuclear Opalescence; NC, Nuclear Color; C, Cortical cataract; P, Posterior subcapsular cataract; OU, Oculi Uterque; IOP, Intraocular Pressure; MD, Mean Deviation; ff-ERG, full-field Electroretinography; HET, Heterozygosis; FSM, Frame-Shift Mutation; HEM, Hemizygosis; PM, Pathologic Myopia; RP, Retinitis Pigmentosa.
